# RECK expression is associated with angiogenesis and immunogenic Tumor Microenvironment in Hepatocellular Carcinoma, and is a prognostic factor for better survival

**DOI:** 10.7150/jca.56167

**Published:** 2021-05-05

**Authors:** Zhao-Ru Dong, Zhi-Qiang Chen, Xiao-Yun Yang, Zi-Niu Ding, Kai-Xuan Liu, Lun-Jie Yan, Guang-Xiao Meng, Ya-Fei yang, Yu-Chuan Yan, Sheng-Yu Yao, Chun-Cheng Yang, Xu-Ting Zhi, Tao Li

**Affiliations:** 1 Department of general surgery, Qilu Hospital, Shandong University, Jinan 250012, China.; 2 Department of Gastroenterology, Qilu Hospital, Shandong University, Jinan 250012, China.

**Keywords:** hepatocellular carcinoma, RECK, angiogenesis, immunotherapy, immune checkpoint inhibitors

## Abstract

Angiogenesis and immunosuppression have been described as closely related processes that can occur in parallel. As an inhibitor of matrix metalloproteinase, whether the level of reversion-inducing cysteine-rich protein with Kazal motifs (RECK) in hepatocellular carcinoma (HCC) reflects a link between angiogenesis and immunosuppression is still unknown. We analyzed RNA expression, immune infiltration and survival of HCC from The Cancer Genome Atlas databases. Immune scores and stromal scores were calculated based on the ESTIMATE algorithm to quantify the immune and stromal components in HCC. The association between RECK and clinicopathological features was further investigated by immunohistochemistry on tissue microarray. We found that the prognosis of patients with high RECK expression was significantly better than that of patients with low RECK expression. High RECK expression was associated with high ESTIMATE Score, recruitment of more tumor-infiltrating lymphocytes, low tumor purity, and high PD-L1 expression. In addition, positive RECK expression was associated with a lower incidence of vascular invasion and recurrence, a lower level of alpha fetoprotein (AFP) and microvessel density and a better tumor differentiation. Multivariate analyses revealed that reduced RECK expression was an independent prognostic factor for recurrence and poor prognosis. In conclusion, high RECK expression reflects an immunogenic and hypovascularity status in HCC. RECK is a promising prognostic marker for survival of HCC and may act as a complementary indicator for patients to receive anti-angiogenic therapy or immunotherapy.

## Introduction

Hepatocellular carcinoma (HCC) is the most common primary liver malignancy and remains a major public health challenge because of the high mortality rates worldwide 1, 2. Over the past decade, the treatment model for HCC has evolved considerably 3. As HCC traditionally occurs in chronically inflamed livers, this inflammation aids to drive oncogenesis and often renders these lesions to be immunogenic 4. Given its high vascularization and immunogenicity, antiangiogenics and immune checkpoint inhibitors (ICI), are two therapeutic approaches that have shown efficacy in HCC 5-7. In addition, combination of these two therapies may be synergistic. Various angiogenic factors-especially VEGF-have been shown to be associated with a range of immunosuppressive effects in the cancer-immunity cycle, such as antigen presentation, T cell priming, T cell trafficking, and T cell tumor infiltrationcan 8. Angiogenic factors can not only bind their cognate receptors expressed by immune cells to suppress their proliferation and cytotoxic function, but can increase regulatory T (Treg) cell proliferation and homing to tumor tissues 8. Anti-angiogenic drugs targeting pro-angiogenic molecules can increase survival of cytotoxic T lymphocytes and decreases Treg cells recruitment to boost antitumour immune responses 6. In addition, anti-angiogenic drugs can also normalize and remodel the tortuous tumor vasculature, enabling efficient tumor infiltration by effector immune cells and resulting in a more favorable immune microenvironment for ICI antitumoral activity. However, response rates of HCC to combination treatment of antiangiogenics and ICIs is only limited to 20-50% of patients across indications. Therefore, identify biomarkers with predictive value for both clinical outcome and response to combination therapies is becoming critical to spare patients from potentially life-threatening toxicity in the absence of clinical benefit 9, 10.

The reversion-inducing cysteine-rich protein with Kazal motifs (RECK) gene is a relatively newly discovered gene with important implications in cancer biology 11. It was first isolated by an expression cloning strategy designed to isolate human cDNAs inducing flat reversion in a v-Ki-ras-transformed NIH3T3 cell line 12. RECK encodes a membrane-anchored glycoprotein which contains serine protease inhibitor-like domains and is associated with the cell membrane through a carboxy-terminal glycosylphosphatidylinositol (GPI)-modification 13. RECK is an important mediator of tissue remodeling and its main function is to inhibit matrix metalloproteinase (MMP)-2, MMP-9 and MT1-MMP post-transcriptionally 13. These MMPs are active in breaking down the extra-cellular matrix (ECM) in both physiological and pathological states, and the balance between ECM breakdown and deposition is critical for endothelial cell homeostasis and contributes to vasculogenesis and angiogenesis 14. RECK keeps these processes under control, via inhibiting MMPs through several mechanisms including direct inhibition of protease activity, regulation of their release from the cell and possibly through sequestration of MMPs at the cell surface 15-19.

The RECK gene was ubiquitously expressed in various normal tissues and non-neoplastic cell lines, whereas in several tumor-derived cell lines and oncogene-transformed fibroblasts, its expression was strongly suppressed 12. Down-regulation of RECK by oncogenic signaling leads to the excessive activation of MMPs, thereby promoting malignant behavior of cancer cells. Restored expression of RECK in cancer cell lines results in strong suppression of invasion, metastasis, and tumor angiogenesis 13, suggesting a role for RECK in the regulation of angiogenesis and tumor malignancy 20. The mechanism behind this, as mentioned above, is thought to be inhibition of MMPs which leads to reduced tissue remodeling and sprouting of vessels 13.

Emerging evidence suggests that antiangiogenic therapies may also have immunomodulatory effects 21. As an inhibitor of angiogenesis, whether the level of RECK in HCC reflects a link between angiogenesis and immunosuppression is still unknown. In addition, the expression of RECK in HCC was not clearly described. Zhang's study revealed that RECK mRNA expression was lower in HCC tissues than that in Non-HCC tissues 22, country to Furumoto's study which revealed that HCC tissues expressed RECK mRNA at the levels even higher than that in the adjacent noncancerous liver tissues 23. In view of these inconsistency and the rare data for RECK expression in HCC, more data are needed to determine the expression of RECK in HCC, and because RECK is known to inhibit MMPs at the post-transcriptional level 13, it is more beneficial to study MMPs protein rather than mRNA levels when looking at the effect of RECK 16.

In this study, we aim to determine the expression of RECK at the protein levels, and analyzed the associations of RECK expression with clinicopathological variables and clinical outcomes of HCC patients. In addition, we want to investigate the possible correlation and interaction of RECK with angiogenesis and immunogenicity in HCC, not only to identify RECK as a potential diagnostic and prognostic marker for HCC, but also identify it as a biomarker with predictive value for immunotherapy and anti-angiogenic therapy.

## Materials and Methods

### GTEx and CCLE analysis

GTEx established a data resource and tissue bank to study the relationship between genetic variation and gene expression in multiple human tissues. Expression of distinct RECK at the mRNA level in different tissues was detected through analysis of the GTEx database (www.gtexportal.org).

The CCLE (Cancer Cell Line Encyclopedia) project is a collaboration between the Broad Institute, the Novartis Institutes for Biomedical Research, and the Genomics Institute of the Novartis Research Foundation to conduct a detailed genetic and pharmacologic characterization of a large panel of human cancer models. The CCLE provides public access to genomic data, analysis and visualization for over 1100 cell lines.

### TCGA data sources

The RNA-sequencing (RNA-seq) dataset of RECK was download from The Cancer Genome Atlas (TCGA) (http://portal.gdc.cancer.gov/) for expression analysis. Fold change was defined as 1.5 and *p*-value was set as 0.05.

### ESTIMATE and TIMER

ESTIMATE (Estimation of STromal and Immune cells in MAlignant Tumor tissues using Expression data) is an algorithm that takes advantage of the unique properties of the transcriptional profiles of cancer samples to infer tumor cellularity as well as the different infiltrating normal cells. ESTIMATE algorithm is based on single sample Gene Set Enrichment Analysis and generates stromal and immune scores to predict the level of infiltrating stromal and immune cells and these form the basis for the ESTIMATE score to infer tumor purity in tumor tissue 24.

ESTIMATE generates three scores: StromalScore (that captures the presence of stroma in tumor tissue); ImmuneScore (that represents the infiltration of immune cells in tumor tissue); EstimateScore (infers and negatively correlates with tumor purity). ESTIMATEScore was the sum of ImmuneScore and StromalScore denoting the comprehensive proportion of both components in TME 25.

TIMER (http://timer.cistrome.org/) is a comprehensive resource for systematical analysis of immune infiltrates across diverse cancer types. This webserver provides robust estimation of immune infiltration levels for TCGA or user-provided tumor profiles using six state-of-the-art algorithms. It provides four modules for investigating the associations between immune infiltrates and genetic or clinical features, and four modules for exploring cancer-related associations in the TCGA cohorts. Each module can generate a functional heatmap table, enabling the user to easily identify significant associations in multiple cancer types simultaneously.

### Correlation analysis of RECK gene expression and tumor mutational burden (TMB), microsatellite instability (MSI), neoantigen count (NC), and immune checkpoint genes

The dataset used comprised mRNA-seq data from TCGA LIHC tumors (https://tcga-data.nci.nih.gov/tcga/). We used Spearman's correlation analysis to describe the correlation between quantitative variables without a normal distribution. A p-value of less than 0.05 was considered statistically significant.

### Human tissue microarray and immunohistochemistry

Tissue microarray (TMA, Shanghai Outdo Biotech Company) containing 297 pairs of HCC tumors from patients who underwent curative resection was used in this study. We obtained approval for this retrospective study from the ethical committee of Qilu Hospital, Shandong University. Curative resection was defined as complete macroscopic removal of the tumor without exposure of tumor cells on the cut surface with macroscopic tumor clearance confirmed on a computed tomography (CT) scan or magnetic resonance imaging (MRI) study of the liver 1 month after hepatic resection 26. Tumor staging was determined according to the TNM classification system of the 8th edition. The histological grade of tumor differentiation was assigned by the Edmondson grading system.

The TMA sections were used for immunochemistry staining. Monoclonal antibodies against human MMP2 (1:100), MMP9 (1:100), RECK (1:100) were purchased from DakoCytomation, Denmark. Immunohistochemistry was carried out using a two-step protocol (Novolink Polymer Detection System, Novocastra, Newcastle, UK) as previously described 27. Monoclonal antibodies against human PD-L1 (1:100) was purchased from Abcam, Cambridge, UK.

### Evaluation of immunohistochemical variables

For immunohistochemistry staining, five fields of approximately 500 cells from each tumor were counted independently by 2 pathologists. MMP-2, MMP-9, RECK, and CD34 staining were reported separately according to the German semiquantitative scoring system. Briefly, depending on the percentage of staining intensity, the staining was classified into 4 groups: (no staining = 0; weak staining = 1; moderate staining = 2; and strong staining = 3) and the percentage of stained cells (0% = 0; 1%-25% = 1; 26% 50% = 2; 51%-75% = 3; and 76%-100% = 4). Final immunoreactive scores were determined by the formula: overall scores = intensity score × percentage score. The overall score ≤ 3 was defined as negative, >3 as positive.

PD-L1 expression was assessed in HCC cells as previously described 28. For neoplastic cells, the percentage of cells displaying unequivocal membranous staining was recorded, and tumors with >1% of positive cells were classified as positive.

### Follow-up

Patients were followed regularly in the outpatient clinic and were monitored prospectively for recurrence according to a standard protocol as previously described 29. All patients were monitored prospectively by serum AFP, liver function, ultrasonography and chest X-ray every two months, and contrast CT was performed every 6 months. Bone scan or MRI was performed if localized bone pain was reported. A diagnosis of recurrence was based on typical imaging appearance in CT and/or MRI scan and an elevated AFP level.

### Statistical analyses

The chi-square test or the Fisher exact probability test was used to evaluate categoric variables, and the Student t test was used to evaluate continuous variables. The cumulative overall survival (OS) rate was calculated using the Kaplan-Meier method and was compared using the log-rank test. Overall survival was calculated from the date of resection to the date of death regardless of the cause of death. Recurrence free survival (RFS) rate was calculated from the date of resection to the date when tumor recurrence was diagnosed or from date of the resection to the last visit, if recurrence was not diagnosed, and the patients were censored at the date of death or the date of last follow-up 30.

Statistical analyses were performed using the SPSS statistical software package (version 13.0; SPSS Inc., Chicago, IL). Two-tailed p values <0.05 were considered statistically significant.

## Results

### Expression of RECK in different types of normal tissues, cancer tissues and cancer cell lines

**Figure 1:**
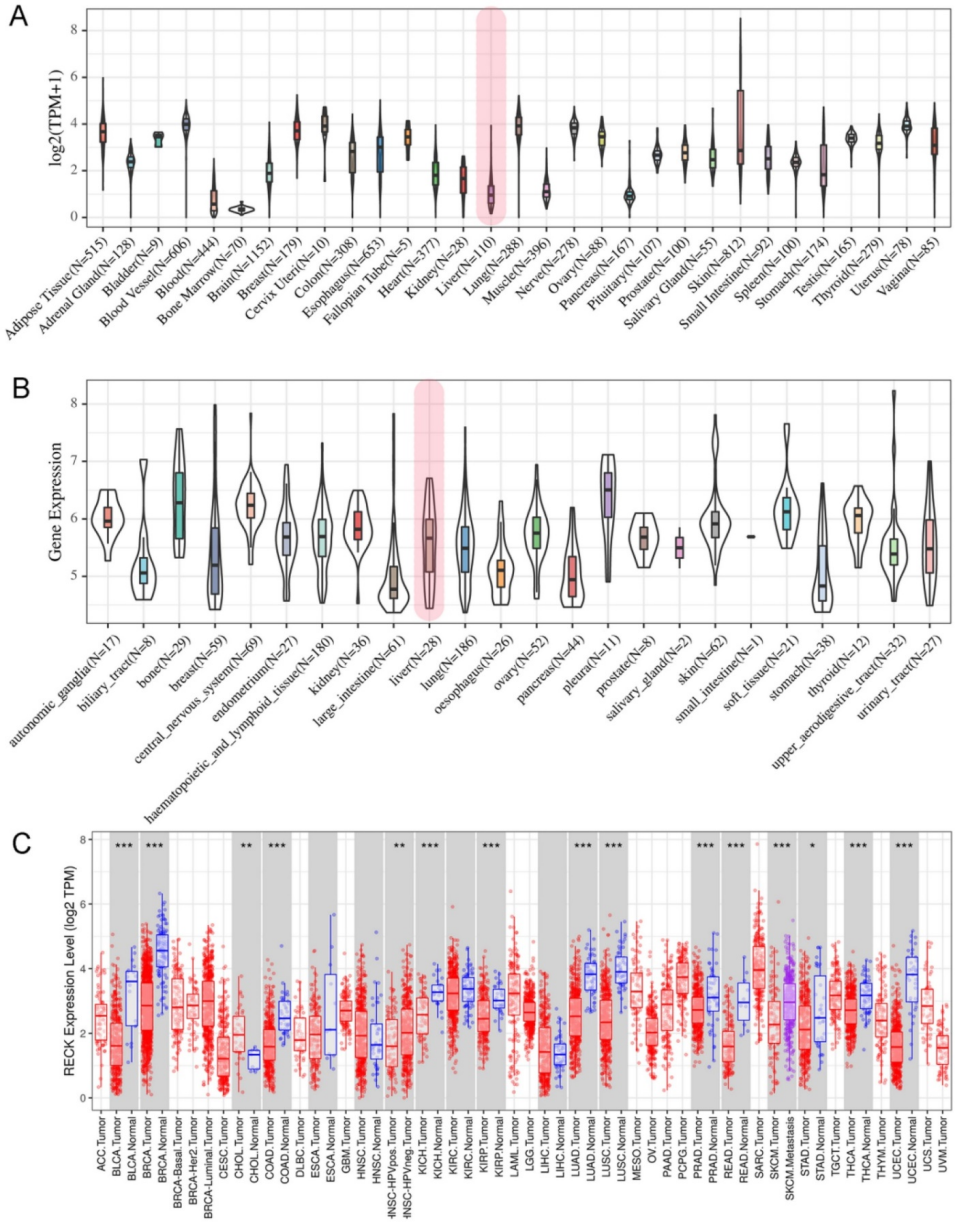
** Expression of RECK in different types of normal tissues, cancer tissues and cancer cell lines.** A: RECK mRNA expression in GTEx database was relatively lower in liver tissues (shown in red frame) as compared to other tissues of digestive system, such as esophagus, small intestine, stomach and pancreases; B:The mRNA expression of RECK in liver cancer cell lines (shown in red frame) from CCLE database was relatively high than most of other digestive cancer cell lines; C:There were no significant differences regarding RECK mRNA expression in HCC tissues and in normal liver tissues from TCGA database. TPM: Transcripts Per Million.

To explore the potential role of RECK in HCC, we first analyzed the expression of this gene by data mining. First, we investigated the mRNA levels of RECK in human tissues using the GTEx database and in tumor cell lines from CCLE database. The results revealed that RECK mRNA expression was lower in liver tissues (Figure 1A, shown in red frame) as compared to other tissues of digestive system, such as esophagus, small intestine, stomach and pancreases. However, the mRNA expression of RECK in liver cancer cell lines (Figure 1B, shown in red frame) was higher than most of other digestive cancer cell lines.

**Figure 2:**
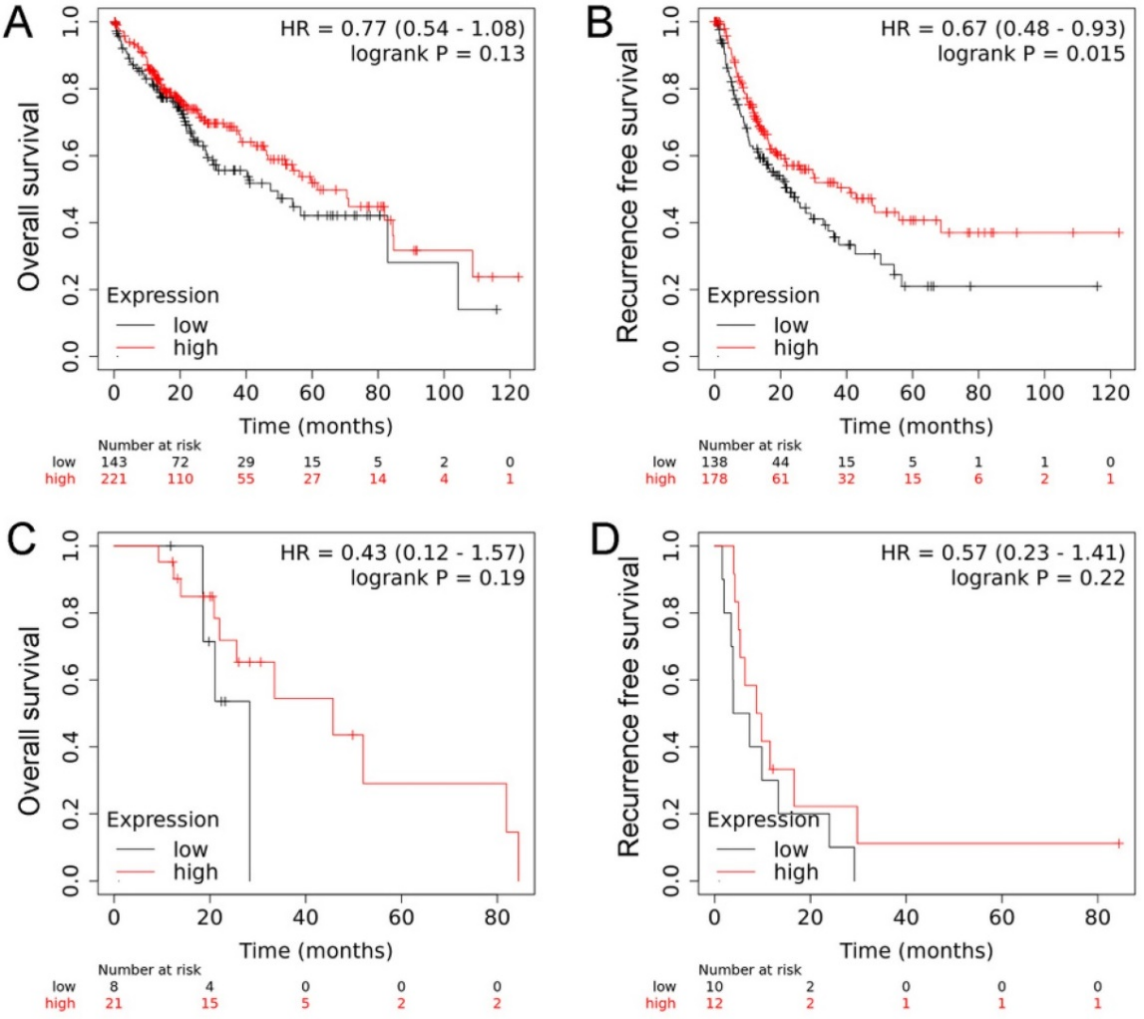
** The mRNA expression on survival of HCC patients from TCGA database.** A: The OS of patients with high RECK mRNA expression tends to be better than that of patients with low mRNA expression, but with no significant difference; B: The RFS of patients with high RECK mRNA expression was significantly better than that of patients with low mRNA expression; C: For patients received sorafenib treatment, the RFS was not significant different between RECK positive and negative patients.

We further investigated the mRNA levels of RECK in cancer tissues using the TCGA database and revealed that there were no significant differences regarding RECK mRNA expression in HCC tissues and in normal liver tissues (Figure 1C). We then investigated the mRNA expression on survival of HCC patients from TCGA database. The OS of patients with high RECK mRNA expression tends to be better than that of patients with low mRNA expression, but with no significant difference (Figure 2A, p=0.130). The RFS of patients with high RECK mRNA expression was significantly better than that of patients with low mRNA expression (Figure 2B). However, for patients received sorafenib (an oral multikinase inhibitor for advanced HCC) treatment (400 mg twice daily), the difference was not significant (Figure 2C, D). Due to limited number of sorafenib-treated cases in this study, further studies are needed to clarify whether patients with low RECK expression will get more benefit from sorafenib treatment.

### RECK expression correlates with immunogenic status in HCC

**Figure 3:**
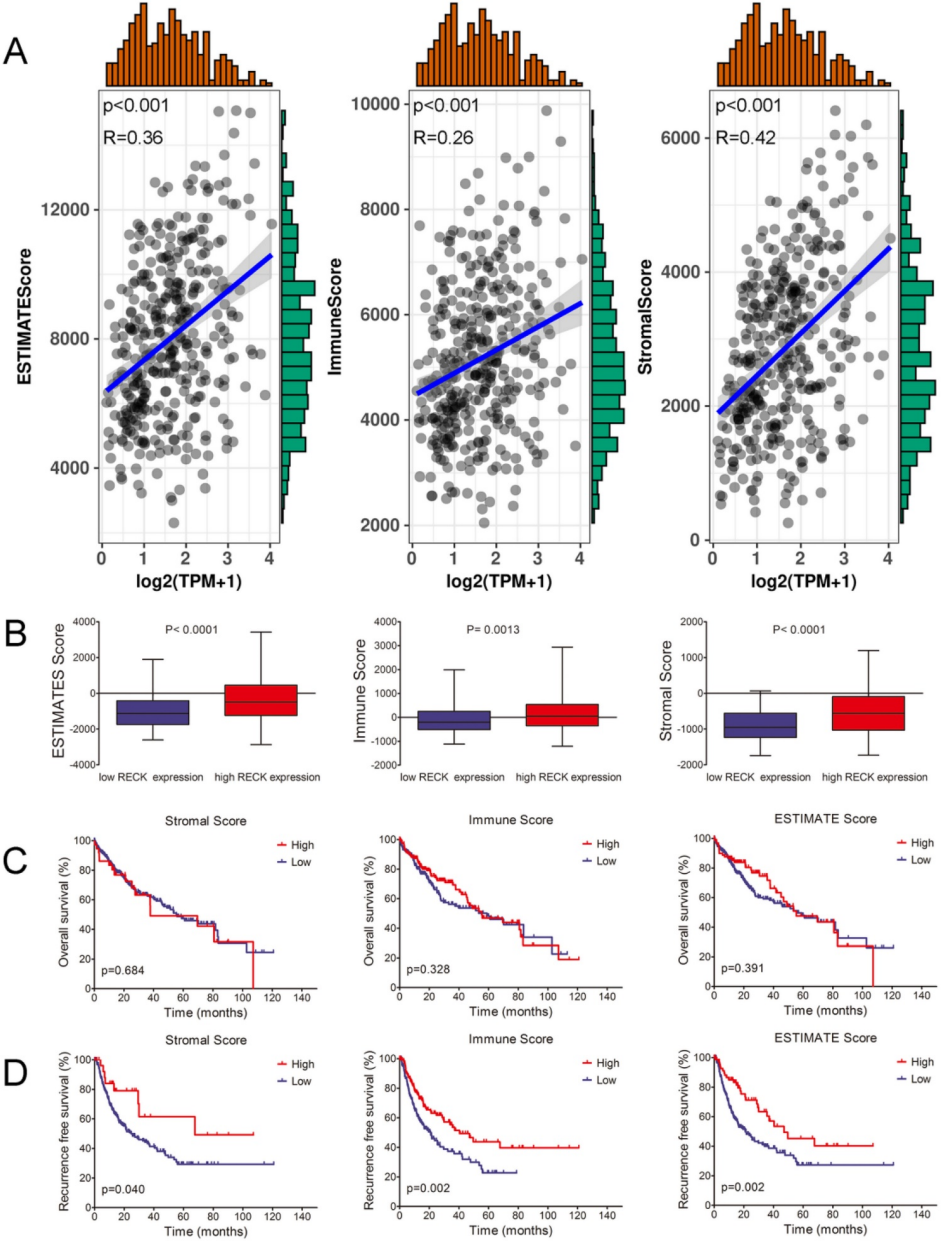
** RECK expression correlates with immunogenic status in HCC.** A: The mRNA expression of RECK was positively associated with ImmuneScore, StromalScore, and ESTIMATEScoreR=0.36, p<0.001). B: The ImmuneScore, StromalScore, and ESTIMATEScore in high RECK mRNA expression group was significantly higher than those of low RECK mRNA expression group. C,D: Though HCC patients with high ImmuneScore, StromalScore or ESTIMATEScore did not show significant better OS than patients with low scores, they did have a significantly better RFS. TPM: Transcripts Per Million.

Emerging evidence suggests that antiangiogenic therapies may also have immunomodulatory effects. As an inhibitor of angiogenesis, whether the level of RECK influence the immune status in HCC is still unknown. By using the ESTIMATE algorithm, we assessed stromal and immune scores, which are represented for the immune or stromal components in tumor microenvironment (TME). As shown in Figure 3A, the mRNA expression of RECK was positively associated with ImmuneScore (R=0.26, p<0.001), StromalScore (R=0.42, p<0.001), and ESTIMATEScore (R=0.36, p<0.001). The ImmuneScore, StromalScore, and ESTIMATEScore in high RECK mRNA expression group was significantly higher than those of low RECK mRNA expression group (Figure 3B), indicating that high RECK expression reflects an immunogenic status in HCC.

To investigate whether the immunogenic status accounts for the better survival of HCC in high RECK group, Kaplan-Meier survival analysis was used for patients with high or low ImmuneScore, StromalScore, and ESTIMATEScore. Though HCC patients with high ImmuneScore, StromalScore or ESTIMATEScore did not show significant better OS than patients with low scores (Figure 3C), they did have a significantly better RFS (Figure 3D). These results implied that the immunogenic status in HCC patients with high RECK expression contributes to a better RFS.

### Correlation between RECK expression and infiltrating immune cells in HCC

**Figure 4:**
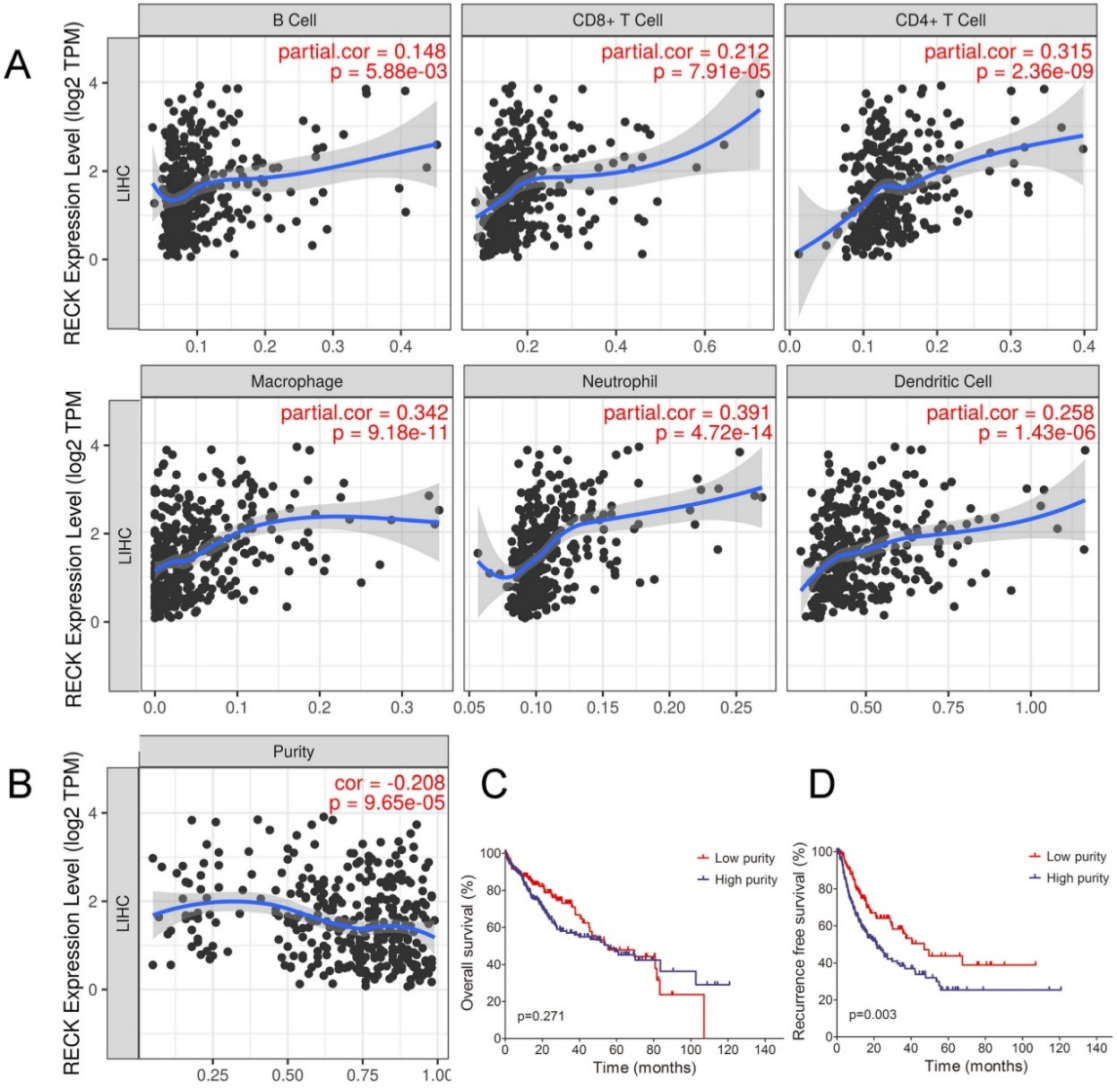
** Correlation between RECK expression and infiltrating immune cells in HCC.** A: Increased expression of RECK in HCC was significantly associated with the infiltration levels of all the 6 immune cells including B cell, CD4+T cell, CD8+T cell, Macrophage cell, Neutrophil cell and DC cell. B: A negative correlation between RECK expression and tumor purity in HCC was found; C: The OS of HCC with low purity was not significantly better than that of HCC with high purity; D: RFS of HCC with low purity was significantly better than that of HCC with high purity. TPM: Transcripts Per Million.

Although the present results showed that RECK mRNA expression correlated with the immunogenic status in HCC, the underlying mechanisms were still unclear. Tumor-infiltrating lymphocytes (TILs) are independent predictors of survival in cancers 31. It is unclear whether upregulation of RECK can lead to the recruitment of more immune cells into the tumor microenvironment and thus affect the prognosis of HCC. We explored the associations between RECK expression and TILs as well as HCC purity by TIMER analysis. As depicted in Figure 4A, increased mRNA expression of RECK in HCC was significantly associated with the infiltration levels of all the 6 immune cells including B cell, CD4+T cell, CD8+T cell, Macrophage cell, Neutrophil cell and DC cell, indicating that RECK mRNA expression is associated the recruitment of more immune cells in HCC. We also found a negative correlation between RECK mRNA expression and tumor purity in HCC (Figure 4B). Though there were no significant differences regarding OS between low purity (high stromal and immune scores) and high purity (low stromal and immune scores) group (Figure 4C), the RFS of low purity group was significantly better than that of high purity group (Figure 4D).

### Correlation of RECK expression with immune subtypes in HCC

**Figure 5:**
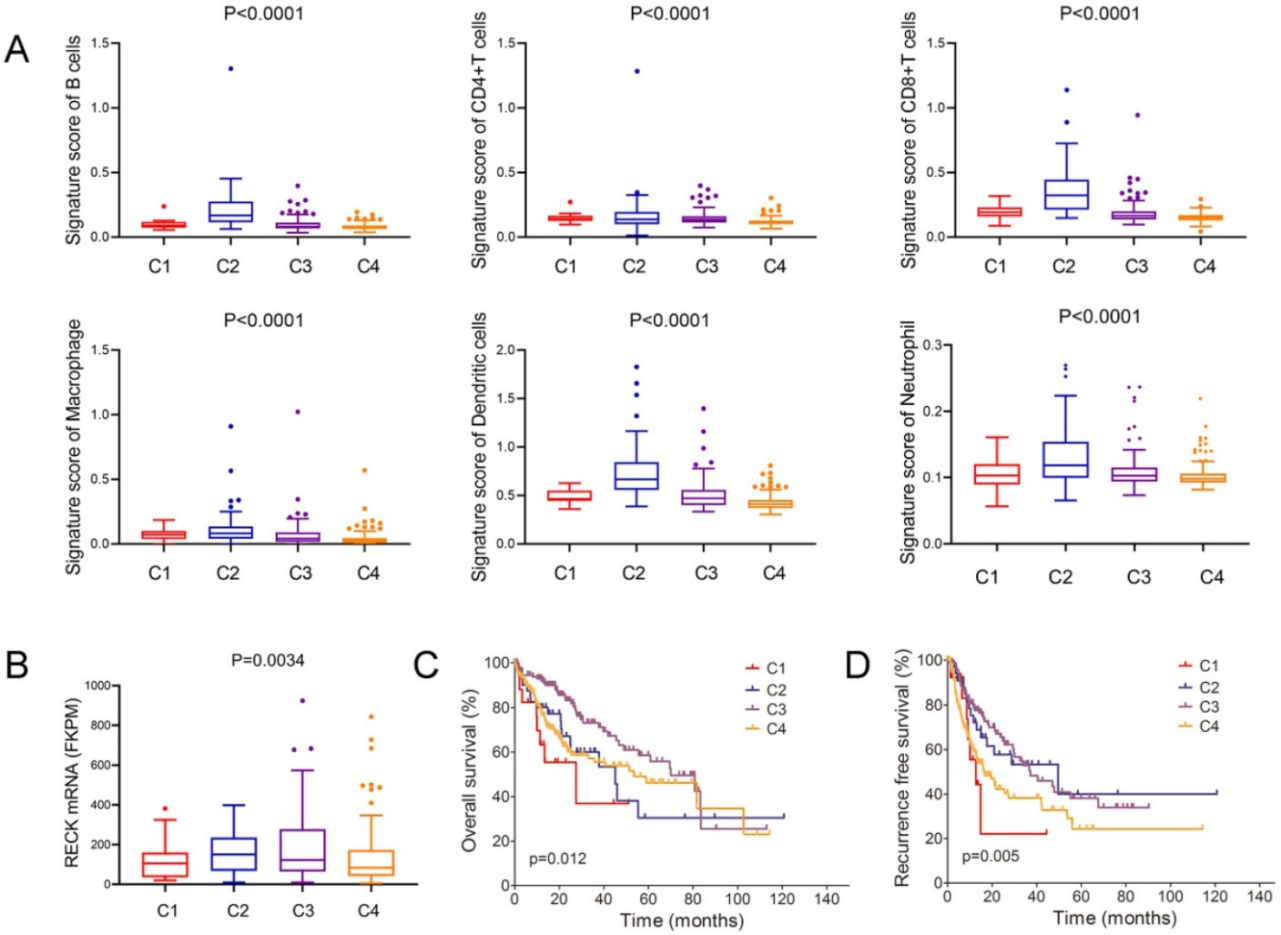
** Correlation of RECK expression with immune subtypes in HCC.** A: C2 type HCC had the highest signature score of all the 6 immune cells than that of other 3 types of HCC; B: C2 type HCC had the highest level of RECK; C, D: C2 type HCC had less favorable OS but had the best RFS.

Thorsson et al. identify six immune subtypes (C1-C6) that encompass multiple cancer types and are hypothesized to define immune response patterns affecting prognosis 32. In this analysis of HCC and comparison of the major four types, we found that C2 type (IFN-γ dominant) had the highest signature score of all the 6 immune cells (Figure 5A). Further analysis of the mRNA level of RECK and immune subtypes revealed that C2 type had the highest level of RECK (Figure 5B). Despite having a substantial immune component, C2 type HCC had less favorable OS but had the best RFS (Figure 5C, D), in accordance with the prognostic role of RECK.

### Correlation of RECK expression with immune checkpoint molecules and angiogenic molecules

**Figure 6:**
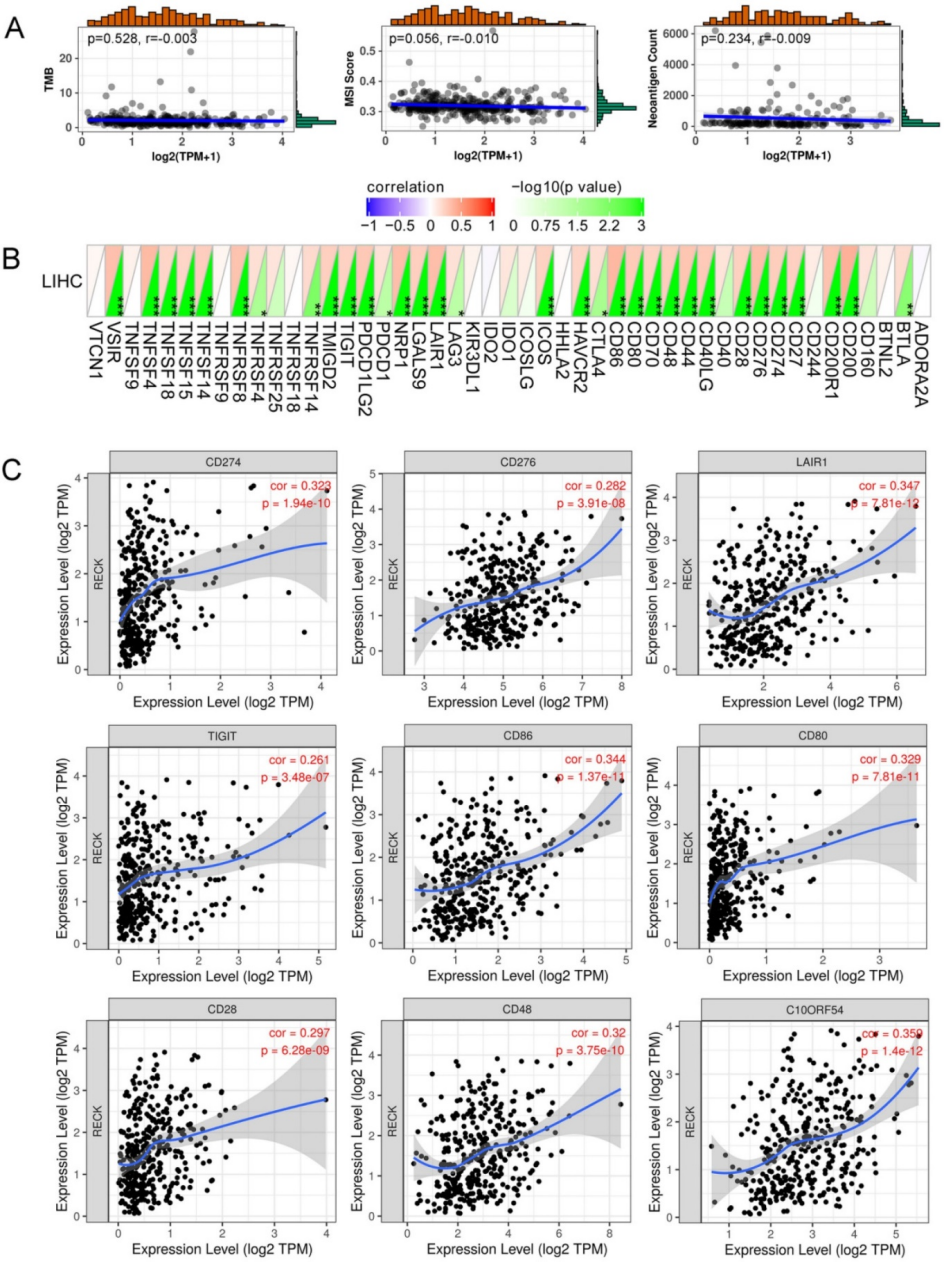
** Correlation of RECK expression with immune checkpoint molecules and angiogenic molecules.** A: RECK expression was not correlated with TMB, MSI and neoantigen count; B,C: the expression of RECK mRNA was significantly correlated with the expression of most immune checkpoint molecules, including PD-L1 (CD274), B7-H3 (CD276), VSIR (C10ORF54), TIGIT, and LAIR-1. TPM: Transcripts Per Million.

Correlation analysis of RECK mRNA expression and TMB/MSI/NC was presented in Figure 6A. RECK expression was not correlated with TMB, MSI and NC. The horizontal axis in the figure represents the expression distribution of the gene, and the ordinate is the expression distribution of the TMB/MSI/NC score. The density curve on the right represents the distribution trend of the TMB/MSI/NC score; the upper density curve represents the distribution trend of the gene. The value on the top side represents the correlation p value and correlation coefficient.

Figure 6B demonstrated a heat map of the correlation between mRNA level of RECK and multiple immune checkpoint genes. The horizontal and vertical coordinates represent genes, and different colors represent correlation coefficients (in the diagram, red represents positive correlation, blue represents negative correlation), and the greener the color represents the two stronger correlation. Asterisks represent levels of significance (*p<0.05, **p<0.01, ***p<0.001). The expression of RECK mRNA was significantly correlated with the expression of most immune checkpoint molecules, including programmed cell death ligand 1 (PD-L1, CD274), B7 homolog 3 (B7-H3, CD276), VSIR (C10ORF54), T cell Ig and ITIM domain (TIGIT), and Leukocyte-associated immunoglobulin-like receptor 1 (LAIR-1) (Figure 6C).

**Figure 7:**
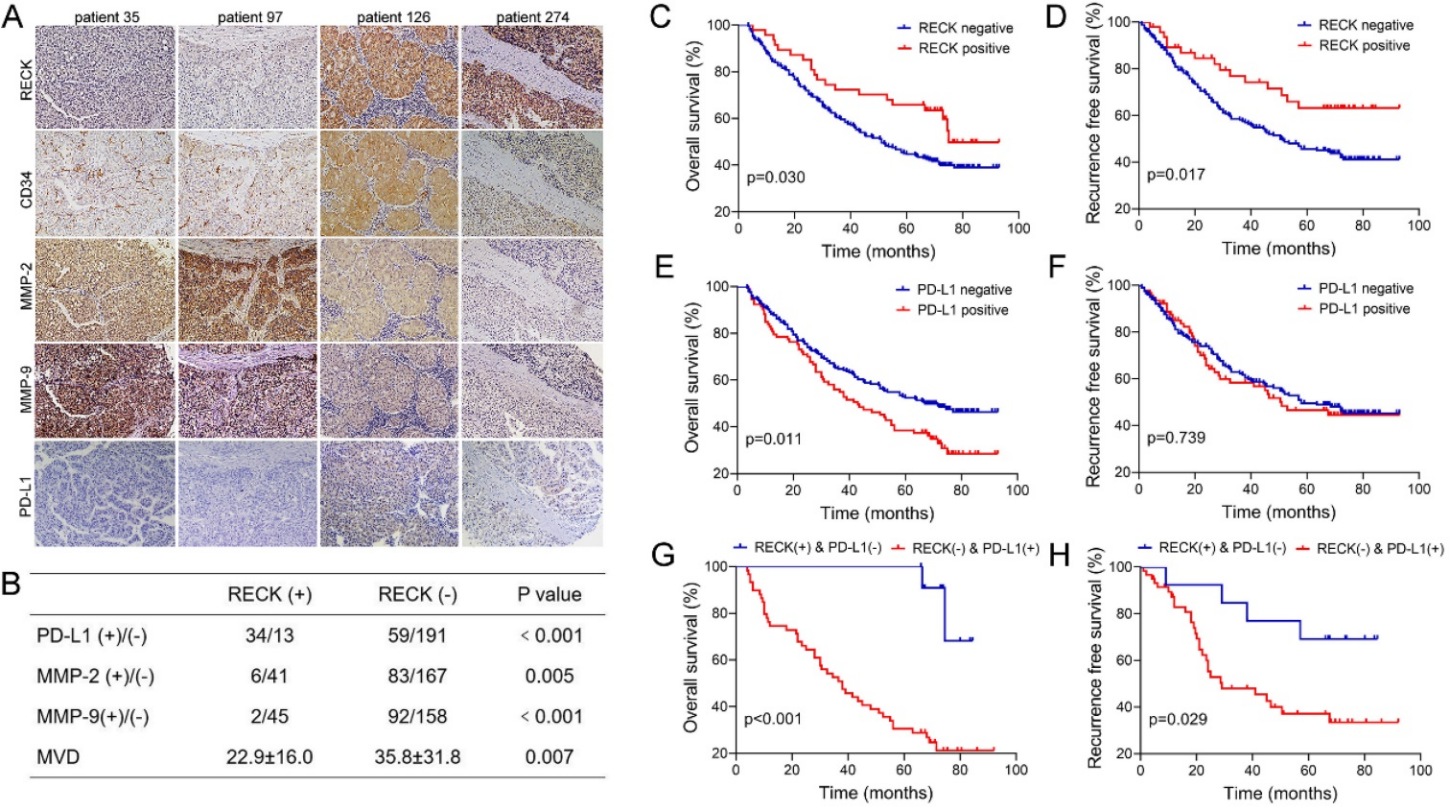
** Expression of RECK or PD-L1 is predictive of prognosis in HCC patients after curative resection.** A: The expression of RECK, MMP-2. MMP-9 and PD-L1 in HCC tissues were assessed by immunohistochemistry staining of human HCC tissue microarray. B: Statistical analysis showed that HCC with positive RECK expression was associated with a significantly higher rate of positive stain for PD-L1, but a significantly lower rate of positive stain for MMP-2 and MMP-9. The MVD in HCC with negative RECK expression was significantly higher than that in HCC with positive RECK expression. C: The OS rates after curative resection for RECK positive patients were significantly better than those of RECK negative patients. D: The RFS rates of RECK positive patients were significantly better than those of RECK negative patients. E: Patients with positive PD-L1 expression had a significantly worse OS than patients with negative expression. F: There was no significant differences regarding RFS. G: The RECK positive and PD-L1 negative patients had the best OS and RFS. F: The RECK negative and PD-L1 positive patients had the worst OS and RFS.

Since PD-L1 expression on tumor cells has been used widely as a predictive marker for efficacy of ICI therapy 33, to further analyze the relationship, the expression of RECK and PD-L1 in HCC tissues were assessed by immunohistochemistry staining of human HCC tissue microarray (Figure 7A). Positive staining of RECK and PD-L1 was detected in 15.8% (47 of 297) and 23.4% (93 of 297) of HCC samples, respectively.

Because RECK is known to inhibit MMPs at the post-transcriptional level, it is better to study MMPs protein rather than mRNA levels when investigating the effect of RECK 34. Therefore, to further analyze the relationship, the expression of MMP-2, MMP-9, and CD34 in HCC tissues were also assessed by immunohistochemistry examination (Figure 7A).

Statistical analysis showed that HCC with positive RECK expression was associated with a higher rate of positive stain for PD-L1 (Figure 7B, p<0.001), and a lower rate of positive stain for MMP-2 and MMP-9 (Figure 7B, p=0.005 & p<0.001, respectively). In addition, the MVD in HCC with negative RECK expression was significantly higher than that in HCC with positive RECK expression (Figure 7B, p=0.007). These results indicate that, in RECK positive patients, ICI treatment may be more efficient because of high PD-L1 positivity, while anti-angiogenic therapy may be less efficient because of low MVD. People should look at the relationship between RECK expression and the efficacy of ICI therapy in future studies.

### Expression of RECK is predictive of better prognosis in HCC patients after curative resection

Though data of RECK mRNA expression from TCGA database revealed only correlation with RFS of HCC patients, however, in our study, univariate (Supplementary Table 1) and multivariate analysis (Table 1) revealed that RECK expression was an independent risk factor for better OS (p=0.001, HR: 0.42, 95% CI: 0.26-0.69) and RFS (p=0.009, HR: 0.49, 95% CI: 0.28-0.84) after curative resection. In contrast, PD-L1 expression was only an independent risk factor for better OS (p=0.009, HR: 1.14, 95% CI: 1.51-3.04). Kaplan-Meier survival analysis revealed that positive expression of RECK is associated with better OS and RFS than patients with negative RECK expression. The 1-, 3-, and 5-year OS rates after curative resection for RECK positive patients were 95.7%,72.3% and 65.8%, significantly better than those of RECK negative patients (84.4%, 60.0% and 44.6%, p=0.030, Figure 7C). The 1-, 3-, and 5-year RFS rates of RECK positive patients were also significantly better than those of RECK negative patients (89.2%, 76.8% and 63.2% vs. 83.2%, 58.0% and 45.7%, p=0.026, Figure 7D).

Our study also revealed that patients with positive PD-L1 expression had a significantly worse OS than patients with negative expression (Figure 7E), but there was no significant differences regarding RFS (Figure 7F). When we evaluated the combined effect of RECK and PD-L1 on the prognosis of HCC patients, we found that the RECK positive and PD-L1 negative patients had the best 1-, 3-, and 5-year OS and RFS (Figure 7G,H, 100%,100% and 100%; 92.3%, 84.6% and 69.2%, respectively) and the RECK negative and PD-L1 positive patients had the worst 1-, 3-, and 5-year OS and RFS (Figure 7G, H, 74.6%, 52.5% and 30.5%; 82.9%, 48.0% and 37.2%, respectively).

### Correlation between RECK, PD-L1, and clinicopathologic characteristics of HCC patients

The correlation between RECK, PD-L1, and clinicopathologic characteristics of HCC patients were investigated, and Table 2 showed the baseline demographic data and tumor characteristics of them. Though there were no significant differences between RECK positive and negative groups regarding gender, age, GGT and ALT level, cirrhosis, tumor capsule, tumor number, tumor differentiation, and tumor size, APF level was significantly higher in RECK negative group than in positive group (p=0.022). Tumors with positive RECK expression had a lower incidence of HBV infection (p=0.008) and vascular invasion (p=0.040), and a better tumor differentiation (p=0.040) than tumors with negative RECK expression. In contrast, PD-L1 expression was only significantly associated with AFP levels (p=0.001), but not associated with other markers of tumor aggressiveness, including tumor number, vascular invasion or tumor differentiation.

## Discussion

Earlier observations indicate that the RECK gene is widely expressed in human organs and a variety of common tumors have been linked to RECK down-regulation, which was associated with reduced survival 35. In the minority of tumor samples where RECK levels are normal or elevated, there is generally a reduction in local invasion, metastasis and an improved prognosis 16. Though analysis of HCC revealed no significant differences between HCC and normal liver regarding RECK mRNA levels, HCC patients with high RECK mRNA expression still showed improved survival than patients with low RECK mRNA expression.

We further analyzed the protein levels of RECK by immunohistochemistry staining of human HCC tissue microarray, and the direct correlation of RECK protein with improved OS and RFS was evident. With higher RECK levels identified in tumors, patients had better survival, lower AFP level, low incidence of HBV infection and vascular invasion, and tumors demonstrated better tumor differentiation. In addition, reduced RECK expression was associated with a shorter recurrence-free survival time, and was an independent prognostic factor for worse OS and RFS, suggesting that reduced RECK expression could be used as an indicator to recognize patients with high risk of tumor recurrence and poor prognosis.

Though tumor stage is inversely correlated with RECK expression in some cancer types, and RECK expression is suggested to be used clinically to enable an estimation of the tumor stage and grade at the initial biopsy 36, however, in our study, no correlation was found between RECK expression and tumor size or tumor number. RECK is known to inhibit MMPs at the post-transcriptional level 12, 13, and our study demonstrated an inverse associations between the expression of RECK and MMP-2 or MMP-9 at protein levels. Importantly, the tumors with positive RECK expression in this study had significantly reduced intra-tumor MVD, and there was a significant inverse correlation between RECK expression and the formation of new vessels, presumably via the mediation of MMP-2 and MMP-9, which are important and powerful inducers of angiogenesis 15, 37. These data suggest that loss of RECK is indicative of angiogenesis and may act as an indicator for anti-angiogenic therapy for HCC. Further studies are needed to clarify whether patients with low RECK expression will get more benefit from anti-angiogenic therapy.

Six immune subtypes (C1-C6) that encompass multiple cancer types have been identified and are hypothesized to define immune response patterns affecting prognosis 32. C2 type tumor is IFN-γ dominant with the highest lymphocytic infiltrate, a CD8 T cell-associated signature, highest M1 content, and a high proliferation rate 32. In this analysis of four types of HCC, we found that C2 type HCC had the highest signature score of all the 6 immune cells, suggesting a robust anti-tumor immune response. Though TILs are independent predictors of survival in cancers, C2 type HCC showed the best RFS but a less favorable OS. One explanation for this discrepancy may be that the immune response simply could not control the rapid growth of tumors. Interestingly, C2 type (IFN-γ dominant) had the highest level of RECK, which is also positively associated with the infiltration levels of all the 6 immune cells and is also a prognostic factor for better RFS. These results indicates that, similar to C2 type, high RECK expression is also indicative of anti-tumor immune response and may associated with enrichment of IFN-γ-related signatures, which is a common trait of ICI-responsive malignancies 9.

Though early AFP response was associated with higher treatment efficacy of ICIs for advanced HCC 38, to date, the most extensively studied predictive biomarkers for efficacy of ICI therapy are TMB and PD-L1 expression 33. However, the value of TMB as a predictive marker for efficacy of ICI therapy in HCC is unclear, because the percentage of patients with high TMB was low, and TMB was not correlated with the rate of predicted neo-antigens or expression patterns of immune related genes 33. Therefore, though PD-L1 may not be a binary marker to decide which HCC patients should receive anti-PD-1 therapy 33, PD-L1 expression on tumor cells is used widely as a selective marker for ICI therapy.

Efficacy of ICI therapy relies on the breadth and depth of effector T-cell responses. Enrichment of IFN-γ-related signatures identified through transcriptomic profiling is a common trait of ICI-responsive malignancies 9. Similar to previous studies 33, PD-L1 expression in HCC cells, with a cut-off of ≥1%, occurred in about 20% of HCC patients in our study and is associated with poor prognosis. Moreover, the expression of PD-L1 is significantly higher in RECK positive HCC than in RECK negative HCC. The reason may be that high RECK expression reflects an immunogenic status in HCC with recruitment of more TIL. TIL can up-regulate expression of immune checkpoints such as CTLA-4 and PD-1, and produce cytokines such as IFN-γ, which leads to expression of PD-L1 on tumor cells and other cells, including T cells, within the tumor tissues 39. Since RECK expression is positively correlated with the expression of most immune checkpoint molecules, including PD-L1, RECK may be a beneficial complement to immune checkpoints for predicting ICI therapy efficacy. Future studies are needed to investigate the relationship between RECK expression and the efficacy of ICI therapy.

Angiogenesis and immunosuppression have been described as closely related processes that can occur in parallel. Various angiogenic factors have been shown to be associated with a range of immunosuppressive effects in the cancer-immunity cycle 8. Therefore, anti-angiogenic agents targeting these factors can stimulate an immune response, which can then be exploited to boost antitumor immune responses. Hence, functional inhibition of MMPs by RECK can suppress tumor growth not only through the suppression of angiogenesis but also through immune-related mechanisms. In our analysis, positive RECK expression is a prognostic factor for better survival and positive PD-L1 expression is a prognostic factor for worse survival. When we evaluated the effect RECK and PD-L1 together, we found the predicted range was extended and sensitivity was more improved than with RECK or PD-1 alone, indicating they are complementary to each other in predicting the prognosis of HCC. HCC patients with positive RECK and negative PD-L1 expression had the best prognosis and may not benefit from anti-angiogenic therapy and ICI treatment, while patients with negative RECK and positive PD-L1 expression had the worst prognosis and may be indicated for both anti-angiogenic therapy and ICI treatment. However, because of the low positivity of RECK and PD-L1 expression in HCC, sample size is small for such patients. Larger scale studies are needed and the efficacy of such treatment needs to be further investigated.

Our study is the first one that describes RECK protein expression in such a large cohort of HCC patients. The reduced RECK expression is a key event for HCC progression and may be regarded as a potential prognostic marker for HCC. The present findings also confirm a negative correlation of RECK with angiogenesis, and a positive correlation with PD-L1 expression, indicating that RECK may act as an indicator for both anti-angiogenic therapy and ICI treatment, but larger scale, prospective studies are needed to evaluate its efficacy.

## Supplementary Material

Supplementary table S1.

## Figures and Tables

**Table 1:** Multivariate analysis of risk factors related to OS and recurrence of HCC patients

Variable	HR	95% CI	*p*
**OS**			
***AFP, ng/mL***			
≤20	1		
>20	1.84	1.27-2.65	.001
***Cirrhosis***			
no	1		
yes	1.62	1.03-2.54	.037
***Tumor size, cm***			
≤5	1		
>5	2.06	1.51-2.81	<.001
***Vascular invasion***			
no	1		
yes	2.06	1.48-2.87	<.001
***RECK expression***			
Negative	1		
Positive	0.42	0.26-0.69	.001
***PD-L1 expression***			
Negative	1		
Positive	1.14	1.51-3.04	<.001
**RFS**			
***GGT, U/L***			
≤50	1		
>50	1.72	1.20-2.45	.003
***Tumor number***			
single	1		
multiple	1.91	1.30-2.82	.001
***RECK expression***			
Negative	1		
Positive	0.49	0.28-0.84	.009

OS: Overall survival; DFS: disease free survival; HR: Hazard Ratio. CI: Confidence Interval; GGT: γ-glutamyltransferase; AFP: a-fetoprotein.

**Table 2:** Correlation between RECK, PD-L1, and clinicopathologic characteristics of patient

Variable	RECK			PD-L1		
	Positive (n=47)	Negative (n=250)	*p*	Positive (n=93)	Negative (n=204)	*p*
**Gender**						
male	44	214	0.135	84	174	0.234
female	3	36		9	30	
**Age, yrs**						
≤50	33	123	0.082	56	104	0.139
>50	14	127		37	100	
**HBsAg**						
positive	34	203	0.008	75	162	0.806
negative	13	47		18	42	
**AFP, ng/mL**						
≤20	23	79	0.022	45	57	0.001
>20	24	171		48	147	
**ALT, U/L**						
≤75	42	231	0.483	87	186	0.487
>75	5	19		6	18	
**GGT, U/L**						
≤50	17	103	0.519	34	86	0.362
>50	30	147		59	118	
**Cirrhosis**						
yes	38	205	0.851	80	163	0.205
no	9	45		13	41	
**Tumor size, cm**						
≤5	24	145	0.378	47	122	0.135
>5	23	105		46	82	
**Tumor number**						
single	38	196	0.706	72	162	0.697
multiple	9	54		21	42	
**Tumor capsule**						
yes	30	145	0.456	54	121	0.839
no	17	105		39	83	
**Vascular invasion**						
yes	6	67	0.040	20	53	0.406
no	41	183		73	151	
**Tumor differentiation**						
I-II	38	164	0.040	65	137	0.639
III-IV	9	86		28	67	

ALT: alanine aminotransferase; GGT: γ^-^ glutamyltransferase; AFP: a-fetoprotein.

## Abbreviations

RECK: reversion-inducing cysteine-rich protein with Kazal motifs
HCC: hepatocellular carcinoma
AFP: a-fetoprotein
ICI: immune checkpoint inhibitors
Treg: regulatory T
GPI: carboxy-terminal glycosylphosphatidylinositol
MMP: matrix metalloproteinase
ECM: extra-cellular matrix
CCEL: Cancer Cell Line Encyclopedia
RNA-seq: RNA-sequencing
TCGA: The Cancer Genome Atlas
TPM: Transcripts Per Million
ESTIMATE: Estimation of STromal and Immune cells in Malignant Tumor tissues using Expression data
CT: computed tomography
MRI: magnetic resonance imaging
TME: tumor microenvironment
TILs: tumor infiltrating lymphocytes
TMB: tumor mutational burden
MSI: microsatellite instability
NC: neoantigen count
IFN-γ: interferon-gamma
OS: overall survival
DFS: disease-free survival
ALT: alanine aminotransferase
GGT: γ-glutamyl transpeptidase
