# Supplementary material for: RECK expression is associated with angiogenesis and immunogenic Tumor Microenvironment in Hepatocellular Carcinoma, and is a prognostic factor for better survival
Source: J Cancer. 2021 May 5;12(13):3827–40. doi: 10.7150/jca.56167 (PMC8176247; doi:10.7150/jca.56167)
Supplement: Supplementary file 1 — Supplementary table S1. [file jcav12p3827s1.pdf]

Supplementary Table 1. Univariate analysis of risk factors related to OS and recurrence of HCC patients

	OS			DFS		
Variable	HR	95% CI	p value	HR	95% CI	p value
Gender						
female	1			1		
male	1.00	0.64-1.57	.999	1.01	0.62-1.66	.964
Age,years						
≤50	1			1		
>50	1.29	0.95-1.74	.103	1.27	0.90-1.78	.170
HBsAg						
negative	1			1		
positive	1.46	0.98-2.19	.062	1.19	0.78-1.81	.429
AFP, ng/mL						
≤20	1			1		
>20	1.82	1.29-2.55	.001	1.27	0.89-1.81	.190
GGT, U/L						
≤50	1			1		
>50	1.55	1.13-2.13	.007	1.66	1.16-2.37	0.005
ALT, U/L						
≤40	1			1		
>40	1.22	0.66-2.56	.517	1.81	0.84-3.87	.128
Liver cirrhosis						
no	1			1		
yes	1.63	1.05-2.54	.030	1.70	1.04-2.80	.036
Tumor size, cm						
≤5	1			1		
>5	1.95	1.44-2.65	<.001	1.21	0.86-1.70	0.286
Tumor number						
single	1			1		
multiple	1.74	1.23-2.44	.002	1.89	1.28-2.78	.001
Tumor capsule						
yes	1			1		
no	1.74	1.28-2.35	<.001	1.49	1.06-2.10	.021
Tumor differentiation						
I-II	1			1		
III-IV	1.33	0.97-1.83	.075	1.21	0.84-1.74	.307

Vascular invasion						
no	1			1		
yes	2.50	1.81-3.45	<.001	1.72	1.17-2.63	0.006
RECK expression						
Negative	1			1		
Positive	0.60	0.38-0.96	.033	0.53	0.31-0.90	.019
PD-L1 expression						
Negative	1			1		
Positive	1.50	1.09-2.04	.012	1.07	0.74-1.54	.740

OS: Overall survival; DFS: disease free survival; HR: Hazard Ratio;

CI: Confidence Interval; GGT:γ-glutamyltransferase; AFP: a-fetoprotein;
